# Genetic Basis of Reproductive Isolation in Torrey Pine (*Pinus torreyana* Parry): Insights From Hybridization and Adaptation

**DOI:** 10.1111/eva.70094

**Published:** 2025-03-31

**Authors:** Lionel N. Di Santo, Alayna Mead, Jessica W. Wright, Jill A. Hamilton

**Affiliations:** ^1^ Department of Biological Sciences North Dakota State University Fargo North Dakota USA; ^2^ Department of Genetics and Evolution University of Geneva Geneva Switzerland; ^3^ Department of Environmental Sciences University of Basel Basel Switzerland; ^4^ Department of Ecosystem Science and Management Pennsylvania State University State College Pennsylvania USA; ^5^ Pacific Southwest Research Station USDA‐Forest Service Placerville California USA

**Keywords:** conifers, forest trees, genetic rescue, reproductive isolation, Torrey pine

## Abstract

Tree species are often locally adapted to their environments, but the extent to which environmental adaptation contributes to incipient speciation is unclear. One of the rarest pines in the world, Torrey pine ( *Pinus torreyana* Parry), persists naturally across one island and one mainland population in southern California. The two populations are morphologically and genetically differentiated but experience some connectivity, making it an ideal system for assessing the evolution of reproductive isolation. Previous work has found evidence of heterosis in F1 mainland‐island hybrids, suggesting genetic rescue could be beneficial in the absence of reproductive barriers. Using ddRADseq and GWAS for a common garden experiment of island, mainland, and F1 individuals, we identified candidate loci for environmentally driven reproductive isolation, their function, and their relationship to fitness proxies. By simulating neutral evolution and admixture between the two populations, we identified loci that exhibited reduced heterozygosity in the F1s, evidence of selection against admixture. SNPs with reduced F1 heterozygosity were enriched for growth and pollination functions, suggesting genetic variants that could be involved in the evolution of reproductive barriers between populations. One locus with reduced F1 heterozygosity exhibited strong associations with growth and reproductive fitness proxies in the common garden, with the mainland allele conferring increased fitness. If this locus experiences divergent selection in the two natural populations, it could promote their reproductive isolation. Finally, although hybridization largely reduced allele fixation in the F1s initially, indicating heterosis is likely due to the masking of deleterious alleles, the emergence of reproductive isolation between populations may diminish the longer‐term benefits of genetic rescue in F2 or advanced‐generation hybrids. As Torrey pine is a candidate for interpopulation genetic rescue, caution is warranted where longer‐term gene flow between diverged populations may result in reduced fitness if barriers have evolved.

## 1 Background

Forest trees are well‐known for maintaining signatures of local adaptation among populations even when experiencing high levels of homogenizing gene flow (Cannon and Petit 2020; Janes and Hamilton 2017; Petit and Hampe 2006). This apparent paradox is explained by strong competition and selection at early life stages: deleterious non‐adaptive alleles introduced by migrant individuals or pollen are likely to be removed from the gene pool (Petit and Hampe 2006). In some cases, population differentiation may lead to reproductive isolation and eventually speciation (Andrew and Rieseberg 2013; Hendry 2009; Hendry et al. 2007; Nosil et al. 2009b; Schluter 2001, 2009). However, the evolutionary and genomic mechanisms underlying the shift from local adaptation with gene flow to the evolution of reproductive isolation are poorly documented in trees, particularly conifers (Bolte and Eckert 2020). The prevalence of local adaptation even under high levels of gene flow makes trees a good system for better understanding how and when divergent selection may drive the evolution of reproductive isolation, a prerequisite for speciation.

Speciation requires the evolution of genetic divergence, which can arise in response to environmental differences, as a result of isolation, or a combination of both (Nosil et al. 2009a, 2009b; Schluter 2001). Allopatry may cause intrinsic reproductive incompatibilities to evolve between populations, resulting in reproductive isolation if they come into secondary contact. However, reproductive isolation can evolve even when gene flow is occurring if selection against migrants and hybrids is strong enough (Schluter 2009). If non‐local alleles are deleterious, selection against them may favor the evolution of reproductive barriers. Extrinsic postzygotic reproductive barriers may occur when environmental selection results in lower hybrid or migrant fitness (as found by Lowry et al. 2008b; Melo et al. 2014; Richards and Ortiz‐Barrientos 2016), while intrinsic postzygotic barriers result in lower hybrid fitness regardless of the environment (Coughlan and Matute 2020). While both types of barriers can be involved in speciation, with great variation in the strength of each type of barrier across systems, extrinsic postzygotic barriers are generally stronger than intrinsic postzygotic barriers (Christie et al. 2022). Additionally, extrinsic postzygotic barriers are stronger in ecotypes than in species, suggesting they may be more important at earlier stages of speciation (Christie et al. 2022) with intrinsic postzygotic isolation being important at later stages, either as a result of divergence or by contributing to reinforcement (Coughlan and Matute 2020).

As reproductive isolation can arise through many mechanisms, including both prezygotic and postzygotic barriers, identifying the genes underlying these reproductive barriers can help us better understand the process of speciation at the genomic level (Choi et al. 2020; Feder et al. 2012; Lowry et al. 2008a; Rieseberg and Blackman 2010; Schluter and Rieseberg 2022; Strasburg et al. 2012). One way to identify mechanisms underlying the evolution of reproductive isolation is through signatures left in the genome (Feder et al. 2012; Nosil et al. 2009a; Schluter 2009; Strasburg et al. 2012). When reproductive isolation is developing amidst ongoing gene flow, as in the early stages of ecological speciation where species diverge as a consequence of natural selection among contrasting environments, loci that are under selection will become more differentiated between two populations than other genomic regions (Feder et al. 2012). As speciation proceeds, variants linked to those regions under selection diverge, eventually leading to differentiation throughout the genome. Analysis of the functions of these divergent regions of the genome can be complemented with phenotypic data where both populations are grown in a common environment. Using genotype–phenotype associations, differentiated loci can be linked to variance in reproductive or fitness‐associated traits. As reproductive‐aged common gardens are uncommon for long‐lived species, our understanding of reproductive barriers and their genomic underpinnings in trees is limited.

To evaluate the genomic and fitness consequences of interpopulation gene flow, we took advantage of a 10‐year‐old common garden with Torrey pine ( *Pinus torreyana* Parry), established in 2007 by the USDA‐Forest Service Pacific Southwest Research Station, which includes reproductive‐aged trees of both parental populations and their F1 hybrids. Torrey pine is one of the rarest pines in the world, with only two native populations remaining (Di Santo et al. 2022). Separated by approximately 280 km (170 miles), the island population native to Santa Rosa Island, one of the Channel Islands, experiences cooler temperatures and greater precipitation on average than the mainland population located at the Torrey Pine State Natural Reserve in La Jolla, California (e.g., mean temperature of the driest quarter is 16.7°C at the island population and 18.9°C at the mainland population; precipitation of the warmest quarter is 17 mm at the island population and 12 mm at the mainland population, Appendices S1 and S2). When grown in a common environment, the two populations exhibit differences in growth, cone, and needle morphology consistent with adaptation to their native environments (Hamilton et al. 2017), but also exhibit relatively low genetic differentiation (*F*_ST_ = 0.0129) and some evidence of ongoing gene flow (Di Santo et al. 2022). In an open‐pollinated common garden including mainland and island trees, the only F1s produced were from island maternal trees fertilized by mainland pollen, with no F1s observed between mainland maternal trees fertilized by island pollen (Hamilton et al. 2017). Asymmetric crossing barriers can be evidence of cytonuclear incompatibilities, or maternal incompatibility such as pollen‐pistil or pollen‐nucellus interactions (Case et al. 2016; Fernando et al. 2005; Tiffin et al. 2001; Turelli and Moyle 2007) and may be a sign that reproductive barriers between the two populations have already begun to evolve (Barnard‐Kubow et al. 2016). Furthermore, fitness metrics measured after 10 years in the mainland common garden environment partly reflect the influence of selection at earlier life stages, so the effect of extrinsic postzygotic isolation may be detected at genetic loci associated with phenotypes. Thus, Torrey pine is an ideal system for investigating the early stages of reproductive isolation arising from extrinsic or intrinsic barriers between two geographically separated populations occurring in contrasting environments.

Understanding the nature of the divergence between Torrey pine populations will inform the species' management. Both populations exhibit exceedingly low genetic diversity, particularly for a conifer (Di Santo et al. 2022; Farjon 2013; Ledig and Conkle 1983), and F1 hybrids between the two populations appear to have higher fitness proxies than the parental populations, suggesting genetic rescue may be beneficial (Hamilton et al. 2017). However, increased F1 fitness may be the result of heterosis, in which excess homozygosity of deleterious recessive alleles is reduced (Tallmon et al. 2004; van de Kerk et al. 2019; Williams and Savolainen 1996). Given this, it is unclear whether increased hybrid fitness would persist in future generations of hybrids backcrossed with either parental population. If the parental populations have evolved partial reproductive isolation as a result of ecological speciation, F2s and backcrossed individuals with incompatible alleles may ultimately have lower fitness despite heterosis in F1s, limiting the value of interpopulation genetic rescue (Christie et al. 2022; Walter et al. 2020). Testing for evidence of reproductive isolation is an important first step to understanding the potential effect of genetic rescue for this species.

In this paper, we investigate whether there is evidence for incipient ecological speciation between Torrey pine populations by testing whether loci with reduced admixture in F1 hybrids are associated with fitness‐related phenotypic differences. We address three main questions: (1) Are there genomic signatures of reduced admixture between the two Torrey pine populations? (2) If so, are alleles with reduced admixture linked to phenotypes or functions that indicate they may be involved in local adaptation and underlie extrinsic postzygotic isolation? (3) What are the implications for conservation of this endangered species—would genetic rescue be beneficial or should these two populations be managed as separate taxonomic groups?

## 2 Methods

### 2.1 Multigenerational Common Garden

Prior to 1960, two plots of 20 mainland and 20 island Torrey pine trees were established adjacent to each other at the USDA Horticultural Field Station (now the Scripps Institute) in La Jolla, CA (Haller 1967; Hamilton et al. 2017). In 2004, open‐pollinated cones from 10 mainland and 16 island trees were sampled in the plots, yielding a total of 643 seeds. In 2006, seeds were planted and grown in a greenhouse at the USDA‐Forest Service Pacific Southwest Station, Institute of Forest Genetics, Placerville, CA. As seedlings emerged, one cotyledon was clipped from each plant for genetic analyses and identification of seedlings' ancestry (island, mainland, or F1 hybrid) using two population‐specific allozyme markers (Ledig and Conkle 1983). Of the 643 seeds planted, 128 were genetically confirmed as being of pure mainland ancestry, 134 were identified as being of pure island ancestry, and 381 were identified as mixed ancestry (F1 hybrids) (Ledig and Conkle 1983; Hamilton et al. 2017). These F1 hybrids solely reflected crossings of island females with mainland pollen donors, indicating hybrids were the product of unidirectional gene flow between parental populations.

In 2007, a subset of 360 seedlings representing each ancestry (*n* = 120 each of island, mainland, and F1 hybrids) was planted following a randomized complete block design with six replicate blocks at the common garden site in Montecito, CA (34.42963, −119.56396). Each of the 360 trees planted was measured for four quantitative traits once a year (Appendix S3), including tree height in cm (measured between 2008 and 2018), the number of conelets or first‐year cones produced (measured between 2013 and 2018), the number of immature cones or second‐year cones produced (measured between 2015 and 2018), and the number of mature or third‐year cones produced (measured between 2015 and 2018). These traits were selected as they provide multiple proxies of fitness, incorporating both growth and reproductive output needed to examine the impact of interpopulation gene flow on Torrey pine fitness (see below). Given that selection likely acts on each reproductive stage and there was limited correlation across traits over time (see below), we analyzed each cone production trait separately using all available years. For full details about the common garden experiment, see Hamilton et al. (2017).

To evaluate whether the measured traits may respond to selection, we estimated narrow‐sense heritability for each trait. We estimated narrow‐sense heritability in R using the function marker_h2 from the package heritability (version 1.4, Kruijer and Kooke 2023), which estimates restricted maximum likelihood estimates of genetic and residual variance contributing to variance in the phenotypic data. We estimated variance resulting from genetic relatedness among individuals using the genetic relatedness matrix (GRM), described below, and included year of measurement and block as covariates.

Adaptation to climate differences between population localities may contribute to extrinsic reproductive barriers. To evaluate the effect of the climate at the common garden environment on the fitness of parental and F1 individuals, we compared climatic variables from the two native Torrey pine populations and the common garden site. We extracted data from climate rasters from *BioClim* version 2.1 for the years 1970–2000 at 30 s resolution (Fick and Hijmans 2017) using the R package *terra*, version 1.7.71 (Hijmans 2024) in R 4.3.2 (R Core Team 2023). The common garden site is a novel environment compared to either native site and is more similar to the island site along PC1 (Appendix S2). However, because the island site generally experiences more moderate conditions (lower temperatures and more precipitation during the warmest quarter, Appendix S1 and S2) and because the severity of the summer drought is the primary stressor in Mediterranean climates and is likely to be a more important factor in adaptation (Granda et al. 2014; Nardini et al. 2014; Riordan et al. 2016; DeSilva and Dodd 2020), we considered the common garden site to be more similar to the native mainland site.

### 2.2 DNA Extraction and Reduced Representation Sequencing

During the summer of 2016, needle tissue was collected from the 262 individuals surviving of the 360 seedlings originally planted within the common garden (Hamilton et al. 2017), including 89 pure mainland, 88 pure island, and 85 F1 hybrid trees. Following collection, needles were dried and maintained on silica gel until genomic DNA could be extracted using approximately 20–35 mg of dried needle tissue based on a modified version of the CTAB protocol (Doyle and Doyle 1987). In this modified version, rough mixing (e.g., vortex) was replaced with slow manual shaking of samples to reduce DNA shearing. The concentration and purity of extracted DNA were evaluated for each sample separately using a NanoDrop 1000 Spectrophotometer (Thermo Scientific). Overall, purity ratios averaged 1.86 and 2.21 for 260/280 and 260/230 respectively, with concentrations of DNA ranging from 153.78 to 4463.31 ng/μL (average = 1138.41 ng/μL). Following extraction, DNA concentration was standardized to 85 ng/μL for all 262 samples, and reduced‐representation sequencing libraries were prepared using the protocol described in Di Santo et al. (2022). Once constructed, all libraries were pooled and sent to the Genomic Sequencing and Analysis Facility (GSAF; Austin, TX) for single‐end sequencing (1 × 100 bp) on 5 lanes of an Illumina HiSeq 2500. Prior to sequencing, the pooled library was size‐selected for fragments within the range of 450–500 bp.

### 2.3 Reference‐Guided Assembly and Calling of Genetic Variants

Raw sequenced libraries were demultiplexed using *ipyrad* version 0.9.12 (Eaton and Overcast 2020). To reduce the probability of assigning a read to the wrong individual, *ipyrad* was parameterized to allow only a single mismatch in the barcode sequence. Raw reads were subsequently filtered for quality using *dDocent* version 2.8.13 (Puritz et al. 2014a, 2014b). Within *dDocent*, using *fastp* (Chen et al. 2018), base pairs with PHRED score below 20 at the beginning and end of reads and Illumina adapter sequences were removed, and reads were trimmed once the average PHRED score dropped below 15. Lastly, reads where more than 50% of base pairs had a PHRED score below 15 were discarded. Cleaned reads were mapped to the *Pinus taeda* (loblolly pine) draft genome version 2.0 (GenBank accession: GCA_000404065.3) using the *BWA‐MEM* algorithm (Li 2013) with default values, except for the mismatch penalty (‐B, default: 4) and the gap open penalty (‐O, default: 6), which were relaxed to 3 and 5, respectively, to account for additional genomic differences that may have evolved between Torrey pine and closely related loblolly pine. Genetic variants were called from sequence alignments using the *SAMtools* and *BCFtools* pipeline (Li 2011). Specifically, we used the multiallelic and rare‐variant calling model (‐m) with no prior expectation on the substitution rate (‐‐prior 0). In total, the pipeline identified 44,493,992 genetic variants, including single‐nucleotide (SNP) and insertion/deletion (INDEL) polymorphisms, that were subjected to quality filtering. First, SNPs and indels with a genotype quality (GQ) < 20 and a genotype depth (DP) < 3 were marked as missing. Next, SNPs and indels with PHRED scores ≤ 20 (QUAL), minor allele counts (MAC) < 3, minor allele frequencies (MAF) < 0.01, genotyping rates across individuals < 0.8, and average depth across individuals > 23 (based on the equation from Li 2014) were filtered out of the raw data. Finally, indels and multiallelic SNPs were removed from the dataset, inbreeding coefficients (*F*_IS_) were summarized across ancestry groups using the *basic. stats()* function implemented within the R package *hierfstat* version 0.5.10 (Goudet and Jombart 2021), and only biallelic SNPs with *F*_IS_ values ≥ −0.5 or ≤ 0.5 were kept. This last filter was applied to account for the large and highly repetitive nature of pine genomes (Grotkopp et al. 2004; Stevens et al. 2016), where paralogous sequences may be misaligned, potentially leading to erroneously called genotypes during SNP calling. Removing SNPs with highly positive or negative *F*_IS_ values may help reduce the proportion of erroneously called genotypes due to the clustering of divergent (negative *F*_IS_) or similar (positive *F*_IS_) paralogous sequences. Of the initial 262 genotyped individuals, 53 were removed from subsequent analyses as they exhibited greater than 50% missing values. In total, 11,379 biallelic SNPs across 209 individuals, including 68 pure island, 75 pure mainland, and 66 F1 hybrid individuals were identified and used for analysis.

### 2.4 Parental Population Hybridization Simulations

To evaluate whether barriers to gene flow may have evolved between Torrey pine populations, we quantified genome‐wide admixture in F1 hybrids, measured as locus‐specific observed heterozygosity (H_O_), specifically searching for SNPs with reduced heterozygosity. Loci with reduced heterozygosity relative to null expectations could indicate incompatibilities between island and mainland alleles that result in negative fitness effects, decreasing the number of surviving heterozygous individuals. A deficit of heterozygous individuals could occur from incompatibilities at any developmental stage, including prezygotic barriers, failed development of embryos or seedlings, or mortality in the common garden environment during the 10 years prior to genetic sampling. Therefore, we take reduced‐heterozygosity loci as evidence of reproductive barriers, but we are unable to determine the developmental stage they act on or fully distinguish between intrinsic and extrinsic barriers here. Using a customized R script (R version 4.1.3) relying on R packages *adegenet* version 2.1.5 (Jombart 2008; Jombart and Ahmed 2011) and *hierfstat*, we simulated the distribution of H_O_ values for each of the 11,379 SNPs expected in the F1 hybrids under the null hypothesis that H_O_ at any locus would be based on the frequency of alleles in the island and mainland populations. To do so, we used a two‐step approach within the function *hybridize()* that simulates hybridization between two populations. First, allele frequencies are derived from genotypes specific to each parental population. Then, hybrid genotypes are generated by sampling gametes in each of these populations using a multinomial probability distribution. SNP‐specific null distributions were produced by repeating this process 1000 times, estimating locus‐specific H_O_ each time from simulated hybrid genotypes using the function *basic.stats()*. To mirror the empirical SNP data set, we simulated 11,379 genotypes for 66 F1 hybrids within each iteration, using allele frequencies derived from 68 and 75 pure island and pure mainland individuals, respectively.

To identify SNPs that may exhibit reduced heterozygosity in F1s relative to expectation, we compared simulated null H_O_ distributions (expected H_O_) with H_O_ values estimated from empirical F1 hybrid genotypes (observed H_O_). For each locus, we computed a one‐sided *p*‐value, defined as the probability that an expected H_O_ value is equal to or lower than the observed H_O_ value at this locus. All *p*‐values were corrected for multiple testing using Benjamini and Hochberg's (1995) False Discovery Rate (FDR) procedure implemented within the R function *p.adjust()*. SNPs that exhibited significantly reduced heterozygosity relative to expectation based on parental allele frequencies (FDR < 0.1) were classified as potential candidate regions of the genome associated with the evolution of barriers to gene flow between island and mainland populations of Torrey pine. Finally, to provide additional support to SNPs identified as exhibiting reduced heterozygosity relative to neutral expectation through simulations, we used the R package *introgress* (Gompert and Buerkle 2010) to compute genomic clines and determine whether SNPs identified with simulations also exhibit nonneutral patterns of introgression. For accuracy, hybrid indices were estimated for all 209 individuals and all 11,379 SNPs using the function *est.h()*, while genomic clines were computed across all individuals only for loci identified as exhibiting reduced heterozygosity through simulations using the *genomic. clines()* function. Significant deviation from neutral patterns of introgression was determined using the permutation test implemented within the function (1000 permutations per locus). Similar as above, *p* values were corrected for multiple testing using Benjamini and Hochberg's (1995) FDR procedure implemented within the R function *p.adjust()*, and SNPs with FDR < 0.1 were considered as significantly deviating from neutral patterns of introgression.

### 2.5 Functional Plant Ontology (PO) and Gene Ontology (GO) Annotation

SNPs exhibiting reduced heterozygosity relative to expectations were functionally annotated to determine whether their functions, processes, or anatomical and temporal expressions may be important to Torrey pine fitness using *blastx* 2.9.0+ (Altschul et al. 1990, 1997; Camacho et al. 2009) and a combination of customized R scripts. First, a region of 5000 bps (5 kbps) from *Pinus taeda* draft genome version 2.0 (GenBank accession: GCA_000404065.3) was extracted 2500 bps before and after each target locus using *samtools* (Li et al. 2009). If a SNP was within the first 2500 bp of a scaffold, the first 5000 bps were extracted. If a scaffold harboring a SNP was shorter than 5 kbps, the whole scaffold length was used instead. Extracted sequences were then blasted against the TAIR10 peptide blastset (TAIR10_pep_20101214_updated). Homology between query and blasted sequences was assessed using *blastx* default parameters, an expectation value threshold of 10^−3^ (‐evalue 0.001), and a maximum number of database hits of 5 (‐max_target_seqs 5). Lastly, mapping of GO and PO terms onto annotated sequences was performed in R using a customized script and GO (ATH_GO_GOSLIM.txt.gz) as well as PO (grow.txt.gz) annotations available online. All databases mentioned throughout this section are available from https://www.arabidopsis.org/download/. While neither conifer‐ nor pine‐specific, the TAIR database provides a more exhaustive set of GO and PO annotations to associate with reduced admixture than is available for conifers. This database remains the most complete with respect to plant gene functions and shares orthologous and homologous sequences with pine that will aid in determining functional categories critical to plant fitness (e.g., Eckert et al. 2010). Functional gene annotation is limited for conifers, particularly for genes involved in reproduction and development (De La Torre et al. 2020). Furthermore, some genes involved in flowering in angiosperms pre‐date their divergence with gymnosperms (Moyroud et al. 2017; Liu et al. 2018; De La Torre et al. 2020). However, because of the evolutionary distance between *Arabidopsis* and Torrey pine, the annotations will be best understood within broad functional categorizations.

Following the same procedure as above, we also annotated 5 kb regions around 500 randomly selected SNPs of the total 11,379 called to investigate whether the set of candidate loci for reproductive isolation was enriched or depleted for specific functions or processes using Fisher's exact test for count data as implemented in the R function *fisher.test()*. For each annotation (GO or PO), we constructed a two‐by‐two contingency table recording the number of successes (the number of times an annotation was observed) and failures (the sum of successes across all annotations minus the number of successes for the annotation of interest) in the candidate and the random set of loci, and tested the null hypothesis of independence of rows (candidate, random) and columns (success, failure). When an annotation was absent from one of the sets of loci, we considered the number of successes associated with that annotation and set of loci to be zero. Resulting *p*‐values were corrected for multiple testing within each annotation data set (GO or PO) and category within annotation data sets (for GO: biological process, molecular function, and cellular component) using Benjamini and Hochberg's (1995) FDR procedure implemented within the R function *p.adjust()*. We assumed an annotation to be either enriched or depleted within a particular set of loci when the FDR associated with the odds ratio for that annotation was inferior to 10% (FDR < 0.1).

### 2.6 Correlation Analysis Between Measured Phenotypes

To limit redundancy across traits and ensure each phenotype largely reflects unique inter‐individual variation, we conducted a repeated measures correlation analysis in R based on temporal measurements taken on all 209 samples using the package *rmcorr* (Bakdash and Marusich 2022). Significance of estimated correlation coefficients was evaluated considering a threshold *α* = 0.05. A correlogram with correlations between all pairs of phenotypes was generated using the R package *corrplot* (Wei and Simko 2021) and is shown in Appendix S4. While some correlation coefficients were significant, no phenotypes were highly correlated (*r* ≥ 0.6). Consequently, all four temporally assessed traits—tree height in cm, the number of conelets (first‐year cones), the number of immature cones (second‐year cones), and the number of mature cones (third‐year cones) produced—were kept for subsequent analyses.

### 2.7 Genome‐Wide Association Analysis (GWAS)

To assess whether genotypic variation at selected SNPs may be associated with variability in phenotypes measured across all available years data (Appendix S3), we conducted a repeated measures genome‐wide association analysis based on all 209 samples and 11,379 genetic variants using the R package *RepeatABEL* (Rönnegård et al. 2016). For each trait, the function *preFitModel()* was used to fit a linear mixed model to the data without including SNP effects to estimate variance components for the trait, including a random polygenic effect (based on a genetic relationship matrix, Appendix S5) and a random permanent environmental effect. Including these effects (computed and assessed internally within the function) accounted for genetic relatedness among individuals (random polygenic effect) and non‐genetic, individual‐specific environmental influences permanently contributing to an individual's trait across years (random permanent environmental effect). In addition, “block” (spatial position of a sample within the common garden) and “year” were considered as random variables within the model. Following this, we used the function *rGLS()*, fitting a generalized least square model to each genetic marker given a covariance matrix (estimated using *preFitModel*), to test for associations between SNPs (considered as fixed effects) and phenotypes while correcting for the effect of random variables. Lastly, SNP‐specific *p*‐values were corrected for multiple testing using Benjamini and Hochberg's (1995) FDR procedure implemented within the R function *p.adjust()*. Of the 11,379 SNPs tested for association with each of the four phenotypic traits, only those with FDR < 0.1 were considered potential candidate markers underlying genotype–phenotype association.

### 2.8 Evaluating Fitness Consequences Following Interpopulation Genetic Mixing

One locus was identified that exhibited both lower F1 heterozygosity than expected under a neutral model and was statistically associated with one or more of the four fitness or fitness‐related phenotypes (hereafter referred to as the shared locus; see Section 3). To suggest whether this locus could be a potential candidate associated with extrinsic reproductive isolation between Torrey pine populations, we required that (1) genotype frequencies for the locus would be biased by population, with each population favoring a different homozygous genotype, and (2) individuals with non‐local or admixed genotypes would exhibit reduced fitness relative to individuals with the more local genotype. As the common garden was planted in Montecito near Santa Barbara, CA, we considered mainland trees as local and island trees as non‐local. To determine whether the frequency of homozygous genotypes (i.e., 0/0 and 1/1) statistically differed between parental ancestries, we counted genotype occurrences across pure island and pure mainland individuals and conducted a Pearson's Chi‐squared test with Yates' continuity correction as implemented in the function *chisq.test()* in R for the resulting contingency table.

A linear mixed model and expected marginal means approach was used to evaluate whether average phenotypes statistically differed among genotypes by leveraging R packages *lme4* (Bates et al. 2015) and *emmeans* (Lenth 2023). For each trait, we built a model where phenotypic variation was explained by the fixed effect of individuals' genotypes at the shared locus, the fixed effect of among‐individual genetic relationships, the random effect of blocks assigned to individuals within the common garden, the random effect of the year the phenotypes were measured, and the random effect of individuals themselves (to account for the repeated nature of response variables). Genetic relationships among common garden individuals were estimated using a principal component analysis implemented within the *dudi.pca()* function from the *adegenet* R package. This analysis was performed on allele frequencies of the full genomic data set (11,379 SNPs across 209 individuals), with missing values replaced by the average allele frequencies across individuals. The first two principal components were used in all four linear mixed models to summarize genetic relationships among individuals, as these components provide good proxies for among‐population (PC1) and within‐population genetic structure (PC2) (Appendix S7).

The *emmeans()* function, computing expected marginal means, was used for the assessment of phenotype average differences among genotypes. Briefly, the function computes genotype‐specific trait averages and standard errors corrected for all effects included within linear mixed models. The function also computes adjusted *p*‐values estimated from t‐ratios calculated between all possible pairwise genotype comparisons. Ultimately, we used this statistic to infer statistical differences in average phenotypes for all traits measured between genotypes. For this analysis, 175 of the total 209 Torrey pine trees present within the full genomic data set were used, as genotypes at the shared locus were missing for 34 individuals.

### 2.9 Evaluating the Distribution of Genetic Variation Across Torrey Pine Ancestries and SNP Sets

The distribution of genetic diversity estimated as observed heterozygosity (H_O_) was compared among pure island, pure mainland individuals, and F1 hybrids for the whole genomic dataset (*N* = 11,379 SNPs), SNPs significantly explaining variation in fitness‐related traits (*N* = 12 SNPs), and SNPs exhibiting reduced F1 heterozygosity relative to expectations (*N* = 185 SNPs). In addition, for each ancestry separately, we quantified the number of loci across the whole genomic dataset that were fixed. A locus was considered fixed within an ancestry when all individuals of that ancestry at that locus were homozygous for either the reference or alternate allele (0/0 or 1/1). H_O_ and numbers of fixed loci were both estimated in R. While H_O_ was computed using the *basic stats()* function implemented within the package *hierfstat*, numbers of fixed loci were computed manually.

To determine whether differences in genetic diversity exist among ancestries within SNP datasets, we performed three Dunn's tests using the package *dunn. test* in R. This test is a nonparametric equivalent of analyses of variance and post hoc tests, as normality for estimated observed heterozygosities within ancestries and SNP sets, assessed either visually or using Shapiro–Wilk normality test, could not be assumed. We accounted for multiple testing by correcting *p*‐values associated with each comparison using Benjamini and Hochberg's (1995) FDR procedure. Two H_O_ averages were considered significantly different when the FDR dropped below 10% (FDR < 0.1).

Finally, Fisher's exact test for count data as implemented within the function *fisher. test()* in R was used to compare the number of loci that are homozygous for either reference or alternate alleles between mainland, island, and F1 individuals. We first leveraged a three by two contingency table recording the number of successes (the number of fixed loci) and the number of failures (the total number of loci minus the number of fixed loci) across all three ancestries, testing the null hypothesis of the independence of rows (island, mainland, F1 hybrid) and columns (success, failure). In addition, we used three two‐by‐two contingency tables of the number of successes and failures (defined as above) to test the null hypothesis of the independence of rows (F1 hybrid, island; F1 hybrid, mainland; island, mainland) and columns (success, failure). The *p*‐value associated with each pairwise comparison was corrected for multiple testing using Benjamini and Hochberg's (1995) FDR procedure implemented within the R function *p.adjust()*. We considered a difference to be significant when the FDR dropped below 10% (FDR < 0.1).

## 3 Results

### 3.1 Trait Heritability

Narrow‐sense heritability varied among the four traits, with the number of conelets having the highest heritability (0.377), followed by height (0.28), number of mature cones (0.266) and number of immature cones (0.133) (Appendix S5).

### 3.2 SNPs Exhibiting Lower‐Than‐Expected F1 Heterozygosity and Their Functional Relevance

Comparison of simulated F1 hybrids between island and mainland individuals for 11,379 SNPs using empirical allele frequencies in each parental population revealed some loci that exhibited a higher degree of homozygosity in the F1s relative to the expectation. A comparison between observed and simulated H_O_ estimates at each locus identified 185 SNPs (FDR < 0.1) with reduced F1 heterozygosity. Interestingly, 181 of the 185 reduced‐heterozygosity SNPs (97.8%) identified with parental population hybridization simulations also were identified as deviating from neutral patterns of introgression through genomic cline analysis, further supporting the nonneutral behavior of these loci. Of the 184 5 kb‐long sequences containing these SNPs (two SNPs were located on the same segment), 75 (41%) could be annotated with the TAIR 10 peptide blastset, with hits in 59 described *A. thaliana* genes. Plant ontology terms could be retrieved for 37 (63%) of the 59 gene hits, while gene ontology terms were retrieved for all 59 gene hits.

**FIGURE 1:**
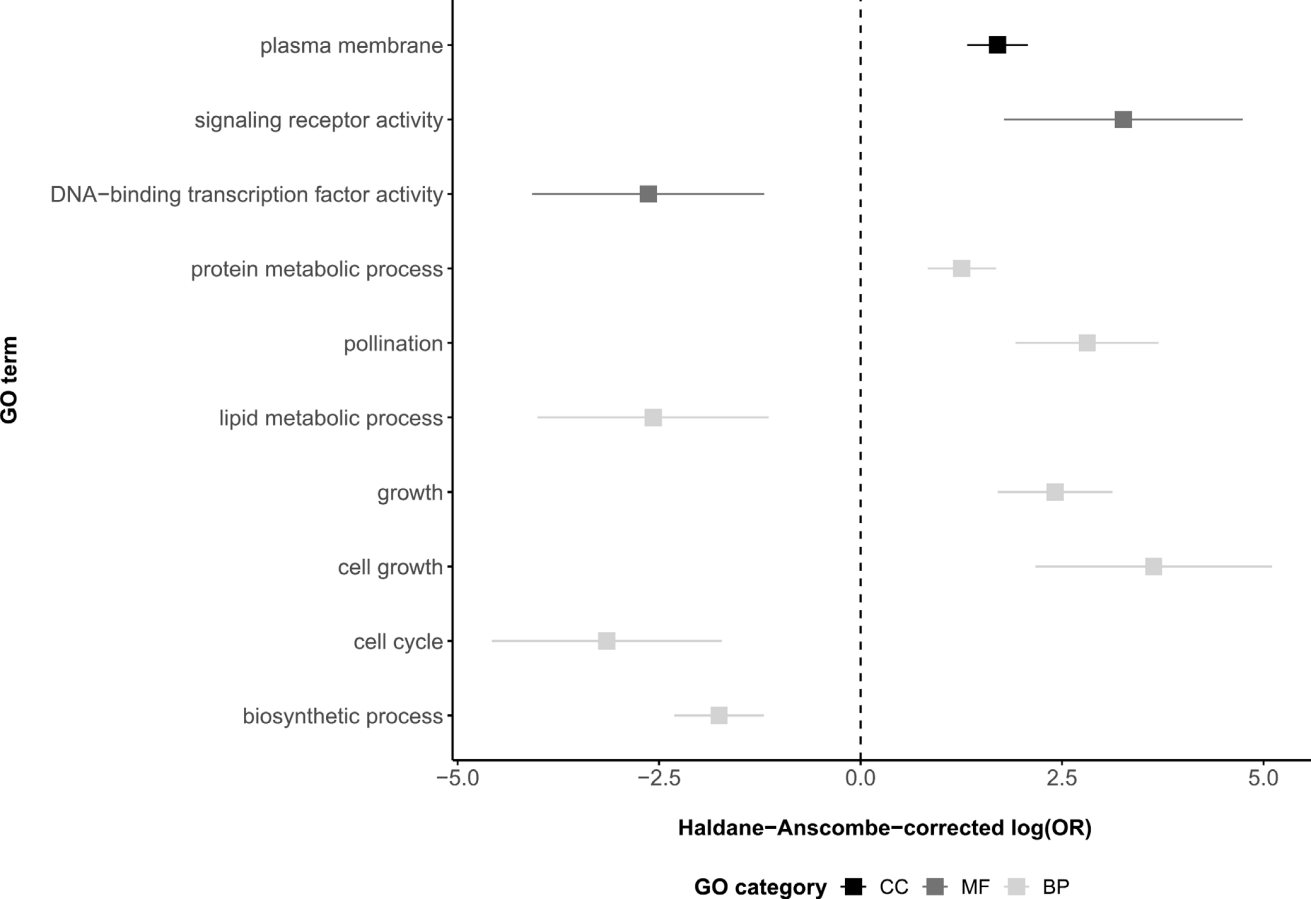
Haldane‐Anscombe‐corrected log‐transformed odd ratios ± standard error (*x* axis) for significantly (FDR < 0.1) enriched (positive values) and depleted (negative values) GO terms (*y* axis) for the set of alleles with reduced F1 heterozygosity, including cellular component (CC, black), molecular function (MF, dark gray), and biological process (BP, light gray) terms. Here, we present Haldane‐Anscombe‐corrected estimates of log‐transform odd ratios as the presence of zero success probabilities within the data would otherwise lead to nonsensical estimates of odd ratios (see Appendix S9 for details).

Regions of the genome that exhibited reduced F1 heterozygosity relative to expectation were associated with a variety of plant developmental stages (Appendix S7). This includes embryo development stages (e.g., C globular stage, D bilateral stage, E expanded cotyledon stage, or F mature embryo stage), vegetative development stages (e.g., 2‐, 4‐, 6‐, 8‐, 10‐, 12‐leaf stages, or seedling development stage), and reproductive development stages (e.g., 4 anthesis stage, petal differentiation and expansion stage, M germinated pollen stage, or L mature pollen stage). Functionally, the majority of these loci are located or active in the nucleus, with molecular functions including protein binding, catalytic activity, RNA binding, as well as kinase and transferase activities (Appendix S9A,B). Biological processes associated with these molecular functions covered responses to various stimuli and stresses, and plant development (Appendix S9C). Enrichment analyses demonstrated that while no PO terms were preferentially associated with SNPs exhibiting lower heterozygosity than expected in the F1, some GO terms were significantly enriched or depleted in the latter SNP set (Figure 1, Appendix S10). Interestingly, assessment of enriched GO terms indicated the products of genes associated with these loci may be preferentially located in the plasma membrane and involved in important developmental and reproductive processes, including cell growth, plant growth, and pollination, with molecular functions associated with signaling receptor activity.

### 3.3 SNPs Exhibiting Statistical Association With Fitness Traits and Their Proxies

Genome‐wide association analysis indicated alleles at several loci may play an important role in contributing to fitness variation in Torrey pine. In total, 12 SNPs were significantly associated (FDR < 0.1) with variation in phenotypes (Table 1). While 8 (67%) of the 12 SNPs were associated with a single trait, the remaining 4 were associated with two or more traits. Phenotypic values of these traits all increased with the number of alternate alleles at associated SNPs except for one, locus_8778 [APFE031129984.1:16200] (Table 1), where they decreased. Interestingly, comparing SNPs associated with fitness and fitness‐related traits with SNPs exhibiting low‐level F1 heterozygosity identified one common locus (locus_4218 [APFE030529380.1:392010]). This locus, in addition to exhibiting a reduced degree of F1 heterozygosity (Appendix S11), was also the only locus that significantly explained phenotypic variation across all four traits measured (Table 1). Unfortunately, functional annotations for the 5 kbps region surrounding this locus were limited. While this region may have functions associated with the mitochondrion (GO:0005739), nothing is known about its molecular function or the biological process it might be involved in. The existence of heterozygotes suggests it is not present in the haploid mitochondrial genome, but may be a nuclear paralog of a mitochondrial gene.

**TABLE 1:** SNPs significantly associated with one or more phenotypes (FDR < 0.1), including tree height (cm), number of conelets, number of immature cones, and number of mature cones. Listed are generic SNP IDs given for this study, SNP IDs retrieved from *Pinus taeda* draft genome (scaffold ID: Position on the scaffold), the trait(s) each SNP is associated with, the effect of increasing the number of alternate alleles on the associated phenotype(s), and the FDR of the genotype–phenotype association.

Generic SNP ID	SNP ID within *P. taeda* genome	Phenotypic trait association	Phenotypic effect	FDR
locus_4218	APFE030529380.1:392010	Tree height (cm)	+	0.047
		Nb conelets	+	< 0.001
		Nb immature cones	+	0.085
		Nb mature cones	+	0.037
locus_12498	APFE031619559.1:67487	Tree height (cm)	+	0.047
locus_433	APFE030047412.1:104020	Nb conelets	+	0.078
		Nb mature cones	+	0.057
locus_3004	APFE030371338.1:302521	Nb conelets	+	0.078
locus_3676	APFE030458498.1:93076	Nb conelets	+	0.024
locus_9630	APFE031249435.1:61968	Nb conelets	+	0.011
		Nb immature cones	+	0.085
		Nb mature cones	+	0.027
locus_13093	APFE031688147.1:172170	Nb conelets	+	0.072
		Nb mature cones	+	0.027
locus_1164	APFE030138697.1:151998	Nb mature cones	+	0.032
locus_6624	APFE030828725.1:2769	Nb mature cones	+	0.027
locus_8019	APFE031020621.1:50406	Nb mature cones	+	0.053
locus_8778	APFE031129984.1:16200	Nb mature cones	−	0.057
locus_10668	APFE031380592.1:30882	Nb mature cones	+	0.053

### 3.4 Fitness Consequences of Hybridization at a Locus Exhibiting Reduced Admixture and Significant Genotype–Phenotype Association

The chi‐squared test performed on homozygous genotype counts for locus_4218 [APFE030529380.1:392010] demonstrated unequal distribution of homozygous genotypes between pure parental ancestries ($\chi_{1}^{2}$ = 104.1, *p* < 0.001, Appendix S11). Homozygotes for the reference allele (0/0) were dominant among pure island individuals (94% of all genotypes) with only two individuals being homozygous for the alternate allele and one being heterozygous, while homozygotes for the alternate allele (1/1) formed the core of pure mainland individuals (97% of all genotypes), with only 2 of 69 mainland individuals being heterozygous. Given this result, we considered the reference allele (0) as the island allele, and the alternate allele (1) as the mainland allele.

**FIGURE 2:**
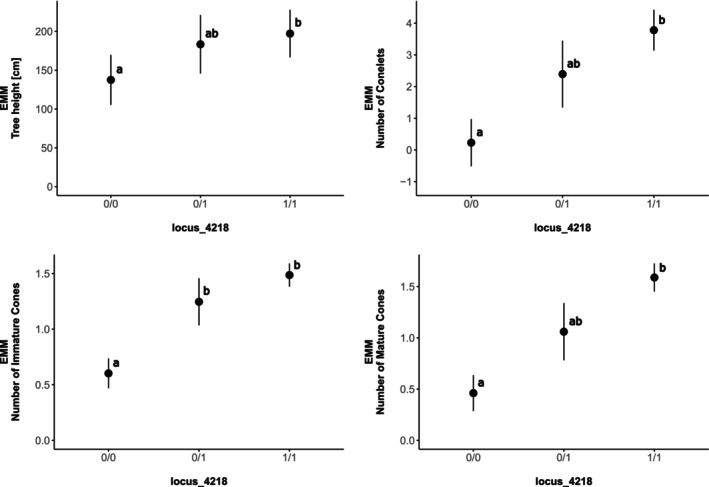
Expected Marginal Means (EMM) ± standard error of all four phenotypes investigated (*y* axis) given the genotype (*x* axis) of island, mainland, and hybrid individuals at locus_4218. Phenotypes measured include tree height (cm), number of conelets, number of immature cones, and number of mature cones. Sample sizes are 47, 5, and 123 individuals with genotype 0/0, 0/1, and 1/1, respectively. Distinct bolded lowercase letters indicate significant differences in phenotypes (adjusted *p* < 0.05) among genotypes (see Appendix S11 for details). Based on genotype frequencies (see Section 3), 0 was defined as the island allele and 1 as the mainland allele.

The evaluation of expected marginal means (phenotype averages corrected for within‐ and among‐ancestry genetic structure, blocks within the common garden, year of trait measurements, and sample ID) revealed unequal fitness across reference, alternate, and heterozygous genotypes. For three out of the four phenotypic traits, individuals homozygous for the island allele exhibited reduced fitness, while individuals homozygous for the mainland allele exhibited greater fitness, with heterozygous genotypes intermediate between the two (Figure 2; Appendix S12). These traits included tree height, the number of conelets produced, and the number of mature cones produced. For the number of immature cones produced, the distribution of fitness among genotypes was slightly different. Individuals homozygous for the island allele still exhibited the lowest fitness, but the fitness of individuals homozygous for the mainland allele and heterozygous individuals was the highest, with no significant differences between groups. We considered the mainland allele (1) as the local, and the island allele (0) as non‐local because both the common garden site and the mainland population experience warmer and drier summers (Appendix S1). Our results thus indicate that carrying at least one copy of the local allele increased tree fitness, with a direct additive relationship based on the dosage of the mainland allele to fitness for tree height (cm), the number of conelets produced, and the number of mature cones produced (Figure 2).

### 3.5 Distribution of Genetic Variation Across Island, Mainland, and F1 Hybrid Individuals

**FIGURE 3:**
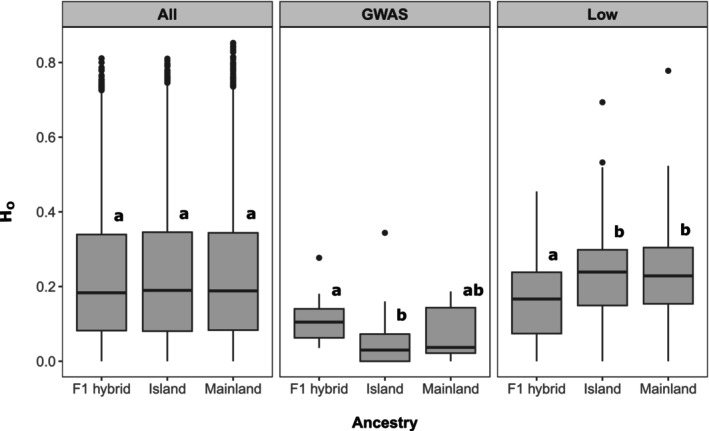
Distribution of observed heterozygosity estimates across ancestries (F1 hybrid, Island, and Mainland) for three SNP sets (All, GWAS, and Low). Distinct lowercase letters indicate a significant difference in observed heterozygosities (FDR < 0.1) among ancestries (see Appendix S12 for details). All: Data set containing all retained SNPs after filtering (11,379 SNPs across 209 individuals), GWAS: Data set containing SNPs exhibiting a significant genotype–phenotype association (12 SNPs across 209 individuals), Low: data set containing SNPs exhibiting reduced heterozygosity relative to expectations (185 SNPs across 209 individuals).

For the whole genomic dataset (*N* = 11,379 SNPs), estimates of observed heterozygosity were similar across ancestries (H_O_ = 0.229, 0.230, and 0.230 for F1 hybrid, island, and mainland individuals, respectively; Figure 3). However, despite almost‐identical average observed heterozygosities, the number of loci homozygous for either the reference or the alternate allele (1/1 or 0/0) varied significantly among ancestries (Fisher's exact test, *p* < 0.001), with island and mainland individuals exhibiting four times a greater number of fixed alleles than that estimated for F1 hybrids (Appendix S12). Overall, 95.3% (614 out of 644) and 93.8% (648 out of 691) of alleles fixed in the mainland and island populations, respectively, varied in F1 hybrids. While these results may seem contradictory, they can be explained by a proportionally higher number of loci with extremely low heterozygosity in the F1s relative to island or mainland individuals (Appendix S14).

Average observed heterozygosity estimated for loci exhibiting reduced F1 heterozygosity for hybrids (0.159) was significantly lower than that for island (0.230) and mainland (0.232) individuals (Figure 3). If they were to contribute to extrinsic reproductive isolation, we would expect heterozygosity at these loci to be low across all three ancestry groups, or at least lower in parental populations than F1s. This pattern would arise due to divergent selection promoting fixation of distinct alleles between island and mainland populations. Additionally, we would expect high genetic differentiation between parental populations at these loci for the same reason. Likewise, if they contributed to intrinsic reproductive isolation, which acts independently of the environment, heterozygosity would be reduced across all three classes. While at least one locus exhibited the anticipated pattern, locus_4218 (*F*_ST_ = 0.933, Appendix S11), other loci displayed low to moderate genetic differentiation between island and mainland populations (Appendix S15), suggesting all reduced‐heterozygosity loci do not contribute to extrinsic reproductive isolation.

For SNPs associated with fitness proxies, we observed exceedingly low genetic variability (Figure 3), with average estimates of heterozygosity for F1, island, and mainland individuals evaluated at 0.113, 0.066, and 0.071, respectively. These results indicate that all three groups have reduced genetic variation available for selection to act upon. However, F1 hybrids did exhibit significantly greater heterozygosity for genetic variants associated with fitness when compared to island individuals. Finally, while elevated genetic differentiation between parental populations is expected if a locus has been under divergent selection, we found that except for locus_4218, other loci contributing to phenotypic variation only were slightly to intermediately differentiated (Appendix S15), suggesting not all SNPs associated with trait variation contribute to local adaptation and extrinsic reproductive isolation.

## 4 Discussion

Local adaptation is common in tree species (Savolainen et al. 2007; Lind et al. 2017, 2018; Gugger et al. 2021), but it is unclear when and how population differentiation may drive the evolution of reproductive isolation. Understanding whether reproductive isolation has developed between two populations is needed to inform species management, particularly where intra‐specific gene flow may be a beneficial source of genetic variation or may be a hindrance, leading to outbreeding depression. In Torrey pine, previous work has shown that the island and mainland populations are genetically and phenotypically different despite some recurrent gene flow, consistent with adaptation to differing environments (Hamilton et al. 2017; Di Santo et al. 2022). Furthermore, asymmetric gene flow between populations when grown together provides some evidence for the evolution of reproductive isolation (Hamilton et al. 2017). Together, this suggests that the two Torrey pine populations could be at an early stage of speciation. Here, we tested for further evidence of the evolution of reproductive isolation by identifying loci exhibiting less heterozygosity in F1 hybrids than expected given allele frequencies in parental populations, suggesting selection against heterozygotes during development or within the mainland common garden environment over 10 years. One locus that was highly diverged among populations and showed signatures of selection against F1 hybrids was associated with fitness differences in the common garden and could be a candidate locus for ecologically driven reproductive isolation between island and mainland Torrey pine populations. Overall, loci with low heterozygosity in F1 hybrids were enriched for functions important in reproduction, such as growth and pollination, suggesting these could be the mechanisms underlying the evolution of reproductive isolation between the two populations.

While demographic modeling has provided evidence for historic speciation with gene flow in pines (Menon et al. 2018; Bolte et al. 2022), the specific molecular mechanisms underlying reproductive isolation in conifers are not well understood (Bolte and Eckert 2020). We identified loci that may be involved in the evolution of reproductive isolation between two populations, including pollination‐ and growth‐related genes, and one locus associated with fitness differences in the common garden consistent with local adaptation to the mainland climate. In combination with asymmetric gene flow, this suggests that both intrinsic and extrinsic barriers could be contributing to reproductive isolation in Torrey pine. These results thus identify potential molecular mechanisms underlying the process of reproductive isolation in a critically endangered pine and suggest that differential adaptation could be a driving force, which is critical information required for informed conservation management of the species.

### 4.1 One Locus Shows Patterns Suggestive of Extrinsic Postzygotic Reproductive Isolation

We identified a locus that is divergent between the two populations and is also associated with fitness differences (locus_4218 [APFE030529380.1:392010]). Individuals with the alternate “mainland” variant of this locus had higher growth and reproduction in the mainland common garden than individuals homozygous for the reference “island” allele, consistent with local adaptation to the hotter, drier environments shared by the mainland population and the common garden environment (Appendices S1 and S2). Torrey pine is likely a relictual endemic species that once had a larger range and became restricted to coastal climates as climate became warmer and drier (Axelrod 1967; Williams et al. 2008), and thus the island population may inhabit a climate more similar to the ancestral climate of the species. Demographic modeling suggests that the two modern‐day Torrey pine populations formed after an ancestral population split, forming a slightly larger island population (*N*_e_ = 2305) and a smaller mainland population (*N*_e_ = 1715) approximately 1.2 million years ago, with gene flow occurring following divergence (Di Santo et al. 2022). It is possible that the mainland population has undergone additional evolutionary change following divergence to adapt to the combined stresses of warm and drier summers associated with the mainland environment, and that this locus is a candidate for local adaptation and divergence of the mainland population. The differentiation between populations for this allele could result from differential selection if heterozygous hybrids have lower fitness in both natural populations relative to individuals that are homozygous for the local variant. If this locus experiences differential selection between the two Torrey pine populations, it could contribute to reproductive isolation. While our results suggest that the alternate allele at this locus may be beneficial in the hotter, drier summers experienced by mainland environments relative to the island population (Appendix S1), a reciprocal transplant of the two populations and their F1 hybrids would be required to determine whether the non‐local variant decreases fitness in both natural populations.

Reduced average heterozygosity at this locus in approximately 10‐year‐old F1 hybrids (0.028 compared to a neutral expectation of 0.936) suggests selection against heterozygotes with one copy of the island and one copy of the mainland allele, or that this locus is linked to or interacts with such a gene under selection. Reduced heterozygosity could result from intrinsic prezygotic or postzygotic barriers (e.g., incompatibilities affecting pollination, germination, or development of seedlings), extrinsic environmental selection in the common garden in the following 10 years, or both. In the common garden, heterozygotes were intermediate in fitness metrics between island homozygotes with lower fitness and mainland homozygotes with higher fitness (although only the number of immature cones was significantly different between island homozygotes and heterozygotes, Figure 2). Our evidence suggests that selection against heterozygotes occurred at early developmental stages, reducing the expected number of heterozygous individuals, and also that the surviving heterozygous individuals in the common garden had intermediate fitness across multiple years of growth and reproduction measurements. This may seem contradictory, but it could indicate that this allele has different fitness effects at different developmental stages, or that pleiotropic interactions with other alleles result in selection against heterozygotes only at certain developmental stages or in combination with specific variants of other alleles.

While there is limited functional annotation for genomic regions proximal to this locus, it does have the annotation “mitochondrion” (GO:0005739). However, as F1 hybrids resulted from unidirectional reproduction between island females and mainland pollen donors, and given the observation that a vast majority of F1s exhibit the homozygous genotypes for the paternal mainland allele (Appendix S11), the locus is likely not encoded in the mitochondrion, which is maternally inherited in conifers. Instead, the “mitochondrion” annotation may arise from ancient paralogy between nuclear and mitochondrial genomes. Alternatively, the locus could be part of a nuclear mitochondrial gene present in the nucleus following mito‐nuclear gene transfer, with its product interacting with mitochondria. Consequently, the locus could be involved in cytoplasmic incompatibility between the maternally‐inherited mitochondrion and nuclear paternal genes (such as those involved in pollen tube growth) (Turelli and Moyle 2007; Rieseberg and Blackman 2010), or with the chloroplast, which is paternally inherited in pines (Neale and Sederoff 1989; Mogensen 1996).

Local adaptation is generally polygenic in forest trees (Yeaman et al. 2016; MacLachlan et al. 2021), suggesting ecological speciation may also have a broad genetic basis (Rose et al. 2018; Schluter and Rieseberg 2022). However, we only find one locus showing evidence consistent with ecological speciation. Because we used ddRADseq data and only sampled a fraction of the genome of Torrey pine, it is likely that other loci underlying reproductive isolation and local adaptation exist, but were not captured in this study. Nonetheless, the fact that we identified a locus linked to both reproductive isolation and fitness despite this limitation suggests that the pattern may be more prevalent throughout the genome and could be better assessed using whole‐genome sequence data.

### 4.2 Functions of Genes Exhibiting Reduced Heterozygosity

If loci exhibiting reduced heterozygosity in F1s are “speciation genes” underlying hybrid incompatibilities, differential adaptation, or both (or linked to involved genes), they should be enriched for related functions (Rieseberg and Blackman 2010; Wright et al. 2013; Walter et al. 2020; Schluter and Rieseberg 2022). Incompatibilities related to pollination or embryo development can influence the evolution of intrinsic barriers, while environmental selection can influence the evolution of extrinsic barriers. Categorizing barriers as solely intrinsic or extrinsic may not reflect scenarios in which both contribute through the interaction of multiple loci (Kulmuni and Westram 2017); however, identifying functional categories associated with loci exhibiting reduced heterozygosity can help identify at which developmental stage barriers may be acting and what processes may be driving them.

We found that highly differentiated loci were located near genes that were enriched for a wide range of functions (Figure 1, Appendix S9). Similar to previous studies, enriched functions included those related to pollination and development (“growth”, “pollination”, “cell growth”) (Rieseberg and Blackman 2010; Leroy et al. 2020). These regions may be involved in intrinsic reproductive barriers, such as pollination incompatibilities associated with female cones or failed embryo development post‐fertilization (McWilliam 1959; Fernando et al. 2005). Other enriched functions include “plasma membrane”, “signaling receptor activity”, or “protein metabolic process”. Given these broad categorizations it is difficult to determine whether these genes could be involved in adaptation to contrasting environments, particularly as only one differentiated gene was associated with fitness differences in the common garden. Previously, island seedlings were found to germinate later and have a reduced growth rate relative to mainland seedlings (Hamilton et al. 2017). The enrichment of loci with the “growth” and “cell growth” GO terms may be related to the evolution of differential growth rates between parental populations. For example, higher growth rates and earlier cone production in the mainland population could be an adaptation to higher fire frequency (Carroll et al. 1987; Hardiman et al. 2016; Schwilk and Ackerly 2001) or lower growth rates in the island population could be an adaptation to lower resource availability (Hamilton et al. 2017). However, it is also possible that the enriched functions are associated with fitness in traits or environmental conditions that were not measured in the common garden. Because the loci with reduced heterozygosity in F1 hybrids were investigated after 10 years of growth in the garden, we cannot determine the stage at which selection against heterozygotes occurred in order to distinguish between intrinsic and extrinsic postzygotic barriers. Crossing experiments and monitoring of hybrids produced between the two populations would provide a more direct approach to determine the developmental stage at which potential barriers act (Christie et al. 2022).

As a group, the identified reduced‐heterozygosity loci were only weakly differentiated among populations (Appendix S15) and had significantly lower Ho only in the hybrid individuals and not in the island or mainland individuals (Figure 3), in contrast to locus 4218. This pattern suggests that for the majority of these loci, the negative fitness effects of heterozygosity are dependent on the genomic background rather than the environment and could mean that these loci interact with genes that are more strongly differentiated between island and mainland populations or with the uniparentally inherited chloroplast or mitochondrial genomes.

### 4.3 Conservation Implications

Similar to previous studies, genome‐wide estimates of heterozygosity for the island and mainland populations were low (Di Santo et al. 2022), suggesting a lack of evolutionary potential. Additionally, we found in the present study exceedingly low diversity at loci putatively underlying trait variation in the species, further supporting these results.

F1 hybrids between mainland and island populations exhibit heterosis, with a higher growth rate and fecundity than either parental population (Hamilton et al. 2017). The four‐fold reduction in the number of fixed alleles when compared to pure island and pure mainland Torrey pine trees, and the almost two‐fold increase in average heterozygosity at loci exhibiting significant genotype–phenotype associations when compared to pure island Torrey pine trees indicates that heterosis in F1 hybrids may result from the masking of deleterious mutations. Furthermore, the narrow‐sense heritability for height and the three reproductive measures fell within the typical range values for growth and reproductive traits in forest trees (Lind et al. 2017), suggesting that the proportion of trait variance that could respond to selection is not significantly reduced compared to other tree species. Alone, these results would suggest that natural populations could benefit from genetic rescue, where hybridization between the two populations could introduce genetic variation to alleviate genetic load. However, our results here warrant caution before introducing non‐local or hybrid trees into the wild. If the natural populations have already developed reproductive isolation or are locally adapted, hybridization may result in decreased fitness that only presents in F2s or later generation hybrids (Lowry et al. 2008a; Walter et al. 2020; Christie et al. 2022), or only in field conditions where they are exposed to stresses not present in the common garden (Melo et al. 2014). Specifically, while some hybrids may still exhibit heterosis, hybrid individuals having incompatible alleles would have decreased fitness, resulting in overall greater variance in fitness which may be harmful to recruitment in natural populations. Future work should use crossing experiments and reciprocal transplants to determine whether genetic rescue could have long‐term benefits to this endangered tree species.

## 5 Limitations

Several possible limitations arise from the common garden experiment. First, it is possible that island individuals were subject to selection pressure in the original 1960s mainland common garden experiment, and that the second‐generation island individuals included in this study are not representative of the genomic makeup of the natural population. However, genetic diversity does not appear to be reduced in island individuals (Figure 3 and Di Santo et al. 2022), and we observe lower fitness in the second‐generation island population than in mainland individuals. This suggests that any possible selection from mortality was not strong enough to eliminate the differences between the two populations or to significantly alter the genomic makeup of the second‐generation island individuals. Second, it is possible that traits measured over 10 years may not reflect lifetime fitness for long‐lived tree species. As we observed slower growth rates in the island population, it is possible that we underestimate their lifetime fitness here. This underscores the importance of long‐term common garden experiments. Together with future experiments, continued monitoring of this second‐generation Torrey pine common garden will continue to inform the conservation of this rare species.

## 6 Conclusions

Taken together, we find evidence of asymmetric barriers to gene flow, reduced heterozygosity in genomic regions related to varying reproductive and developmental functions, and one low‐heterozygosity and highly differentiated locus that associates with reduced fitness in individuals carrying one or two non‐local (island) alleles for all four measured fitness metrics in Torrey pine. These results suggest that Torrey pine populations may be beginning to evolve reproductive isolation, and this may partly be driven by adaptation to contrasting island and mainland environments, in which case introducing non‐local individuals or hybrids to the natural populations for genetic rescue may actually be harmful in the long term. In the future, whole‐genome sequencing combined with reciprocal transplant experiments and the development of experimental crosses including F2s and backcrosses will be poised to identify the genes that may be under divergent environmental selection in the two populations, and/or contributing to the evolution of reproductive isolation (Schluter and Rieseberg 2022). Furthermore, understanding the extent of reproductive isolation among these two populations will inform management strategies for this rare species that balance the benefits of genetic diversity and local adaptation.

## Conflicts of Interest

The authors declare no conflicts of interest.

## Supporting information


Appendices S1–S15

## Data Availability

Genomic and phenotypic data generated for this study, as well as scripts used for analysis, are available from Dryad: https://doi.org/10.5061/dryad.pc866t1xg. https://datadryad.org/stash/share/g_AMBnVOfPuCXNJ‐ic22EWFlZcVBGKJnlx7SPCH98rU.
